# Increased circulating Endothelin-1 is a risk factor for ECMO use and mortality in neonates with congenital diaphragmatic hernia: a prospective observational study

**DOI:** 10.1186/s12931-025-03188-8

**Published:** 2025-03-21

**Authors:** Lotte Lemloh, Aster de Vadder, Tamene Melaku, Bartolomeo Bo, Neil Patel, Stefan Holdenrieder, Andreas Mueller, Florian Kipfmueller

**Affiliations:** 1 Department of Neonatology and Pediatric Intensive Care, Children’s Hospital, University of Bonn, Venusberg-Campus 1, 53127 Bonn, Germany; 2 Department of Neonatology, The Royal Hospital for Children, Glasgow, UK; 3 Institute for Laboratory Medicine, German Heart Centre, Technical University of Munich, Munich, Germany; 4 Department of Neonatology and Pediatric Intensive Care, University Medical Center Mannheim, University of Heidelberg, Mannheim, Germany

**Keywords:** Congenital diaphragmatic hernia, Endothelin-1, Pulmonary hypertension, Extracorporeal membrane oxygenation

## Abstract

**Background:**

Elevated levels of Endothelin-1 (ET-1), a vasoactive peptide, have been associated with adverse outcomes in neonates with congenital diaphragmatic hernia (CDH). However, the relationship between ET-1 levels and clinical outcomes remains poorly understood. This study aimed to investigate the kinetics of ET-1 levels in CDH neonates from birth to 48 h postnatally and assess its association with clinical comorbidities, the need for extracorporeal membrane oxygenation (ECMO), and mortality.

**Methods:**

A prospective single-center study was conducted, including 107 newborns with CDH from 2014 to 2022. Blood samples for ET-1 measurement were collected at birth, 6 h, and 48 h postnatally. The need for ECMO and mortality served as primary and secondary clinical endpoints. Based on the ET-1 values patients were assigned to ET-1 high, intermediate, and low groups. Statistical analyses, including ROC curve analysis and multivariate logistic regression, were performed to determine the predictive value of ET-1 levels.

**Results:**

Among the 107 CDH neonates 41 (38.3%) required ECMO and the overall mortality rate was 19.6%. Higher ET-1 levels at 0 and 48 h correlated significantly with the need for ECMO (*p* = 0.028 and *p* < 0.001) and mortality (*p* = 0.016 and *p* < 0.001). The high ET-1 group had a significantly higher rate of ECMO use (63.2%) and higher mortality (42.1%) compared to the ET-1 low group (15.4% and 0%). Furthermore, elevated ET-1 levels were associated with more severe disease characteristics including severe PH and biventricular dysfunction.

**Conclusions:**

Elevated ET-1 levels during the first 48 h of life in CDH neonates are significantly associated with increased rates of ECMO and mortality. These findings underline the potential of ET-1 as a predictive biomarker for poor outcomes in CDH and highlight its relevance in guiding therapeutic interventions.

**Trial registration:**

DKRS00034329.

## Background

Congenital diaphragmatic hernia (CDH) is a rare neonatal condition affecting approximately 2.3 newborns per 10,000 live births [1–3]. The pathogenesis of CDH is not yet fully understood, but it is believed to result from multifactorial causes including genetic and environmental factors [4]. The diaphragmatic defect allows abdominal organs to migrate into the thoracic cavity compressing the lungs and impairing proper lung development. CDH management continues to evolve, but it frequently necessitates neonatal intensive care unit (NICU) admission and remains the leading indication for extracorporeal membrane oxygenation (ECMO) for respiratory failure in neonates [5]. The mortality rate of CDH neonates is approximately 25–40% [6]. Affected neonates face three primary pathophysiological changes: lung hypoplasia, pulmonary hypertension (PH), and cardiac dysfunction [7]. Endothelin-1 (ET-1), a vasoactive peptide, plays a significant role in the regulation of vascular tone by binding to two receptor subtypes: endothelin receptor A (ET_A_) leading to vasoconstriction and endothelin receptor B (ET_B_) promoting vasodilation [8]. Elevated circulating ET-1 levels have been observed in neonates with PH and most notably, higher plasma levels of ET-1 in CDH neonates are associated with poor outcomes during the first weeks of life [9]. This study aimed to investigate the kinetics of ET-1 from birth until 48 h postnatally and its association with the need for ECMO, mortality, and disease progression in neonates with CDH.

## Methods

### Patients and study design

We performed a prospective single-center (University Hospital of Bonn) subgroup analysis of CDH newborns born between 2014 and 2022, who were initially enrolled in the *“****Bi****omarker for****O****utcome****P****redic****t****ion****i****n Neonates with****C****ongenital Diaphragmatic Hernia”* (BIOPTIC) study (Clinical trial registration with German Clinical Trial Register (DKRS), trial registration number: DKRS00034329; Registration date: 24.05.2024). Patients were eligible for study participation if a sufficient amount of blood plasma was available for ET-1 measurements. Exclusion criteria were severe congenital heart defects requiring surgery or catheter-based intervention within 60 days of life, major non-cardiac congenital anomalies (e.g., omphalocele, lung lesions, chromosomal anomalies), or palliative care. All data were collected from the electronic medical records and the electronic patient charts and documented in our institutional CDH patient database.

### Ethical approval and consent to participate

The study was approved by the Institutional Review Board of the University Hospital of Bonn (local registration number: 047/14). Written informed consent was obtained from parents or legal guardians before study enrollment.

### Neonatal intensive care unit treatment

Newborns with CDH were treated according to a standardized protocol that involved permissive hypercapnia, lung protective ventilation, and delayed surgical repair [10]. Inhaled nitric oxide (iNO) was administered as first-line pulmonary vasodilator, followed by intravenous sildenafil. Bosentan was administered, when PH on echocardiography exceeded 2/3 of systemic pressure, despite the use of iNO or sildenafil, or in the presence of a persistently high oxygen demand. Decisions regarding inotropic support and ventilator settings were made by the attending physician. ECMO was commenced based on specific criteria [11]: preductal oxygen saturation below 85% or postductal saturation below 70%, an oxygenation index of 40 or higher, a PaCO2 exceeding 70 mmHg with a pH lower than 7.15, a peak inspiratory pressure of 28 cmH2O or more, a mean airway pressure of 17 cmH2O or more, and persistent systemic hypotension (mean arterial pressure below 40 mmHg) despite therapy. ECMO was administered using the Deltastream console in combination with a MiniLung petite kit, that contains a DP3 rotational pump with a diagonally streamed impeller, and a Hilite 800 LT oxygenator (Xenios AG, Fresenius Medical Care, Heilbronn, Germany) [12].

### Echocardiographic assessment

Echocardiographic assessment was obtained within the first 12 h of life, at 14 days (± 4 days) of life, and every 7 days if moderate or severe PH was present at 14 days following the pediatric guidelines of the American Society of Echocardiography (ASE) [13]. All studies were performed using a Philips CX50 CompactXtreme ultrasound system equipped with an S12-4 sector array transducer (Philips Healthcare, Best, The Netherlands). Measurements were taken either during the echocardiographic examination or, if postprocessing was required, the studies were digitally recorded and stored for subsequent analysis with offline software (IntelliSpace Cardiovascular, Philips Healthcare, Best, The Netherlands). The severity of PH was classified as mild (pulmonary artery pressure [PAP] < 2/3 systemic systolic pressure), moderate (2/3 systemic pressure to systemic pressure), or severe (suprasystemic pressure) based on the criteria by Keller et al. [9]. The assessment encompassed the ductus arteriosus (DA) flow pattern, the position of the interventricular (IV) septum, and the velocity of the tricuspid regurgitation jet. Cardiac function was evaluated using both qualitative and quantitative methods in a stepwise approach and was categorized as normal, right ventricular (RV) dysfunction (indicated by global or regional hypokinesia, S’ wave < 5.0 cm/s on tissue Doppler imaging, tricuspid annular plane systolic excursion < 0.7 cm, or RV fractional area change ≤ 25%), left ventricular (LV) dysfunction (indicated by global or regional hypokinesia, fractional shortening ≤ 25%, ejection fraction ≤ 45%, or LV output < 100 ml/kg/min), or biventricular dysfunction (a combination of LV and RV dysfunction). Ventricular disproportion was defined as a right ventricular diameter (RV_D_) to left ventricular diameter (LV_D_) ratio (RV_D_/LV_D_) ≥ 1.1. Using an apical 4 chamber view, RV_D_ and LV_D_ were measured directly distal to the tricuspid and mitral annulus as a horizontal line from the endocardium of the RV and LV free wall to the endocardium of the interventricular septum. End-diastole was defined as the frame with the maximum ventricular area corresponding to mitral valve closure and at the end of the R wave on ECG trace [14].

### Endothelin-1 measurements

Blood drawn at birth (0 h) from the umbilical vein as well as at 6 h and at 48 h postpartum from a peripheral arterial line was analyzed. ET-1 levels were measured in pg/mL. The samples underwent immediate centrifugation at 4,000 x g for 10 min at 4 °C. Subsequently, the plasma was stored at -80 °C. ET-1 values were then measured in the local laboratory using ELISA (ET-1-Quantikine, R&D) according to the manufacturer’s instructions.

### Outcomes

Need for ECMO was used as the primary clinical endpoint of this study. Need for ECMO was defined as patients who received ECMO, and patients fulfilling ECMO criteria but with contraindications to ECMO (e.g. low birth weight) who subsequently died within 48 h of life. The secondary clinical endpoint was mortality.

### Statistical analysis

SPSS version 29 (IBM Corp.) was used for data analysis. For the descriptive analysis, continuous variables were summarized as median and interquartile range (IQR). Categorical variables were described as absolute number (n) and percentage. Mann-Whitney-U-test or Kruskal-Wallis-test were used to compare continuous variables between groups and Pearson’s Chi^2^ test and Fisher’s exact test for categorical covariates. ROC analysis was used to determine the area-under-the-curve (AUC) and high and low cut-off values for each timepoint to predict the primary and secondary endpoint. The AUC values for ROC analysis are reported with 95% confidence intervals. Group allocation of patients was based on their outcome (i.e. mortality and the need for ECMO) and the ET-1 values measured. Classification based on ET-1 values was as follows: patients who presented an ET-1 value above the upper cut-off value at one or more measurement points were assigned to the ET-1 high group. Patients who had an ET-1 value below the lower cut-off values at all measurements were assigned to the ET-1 low group. The remaining patients were allocated to the ET-1 intermediate group. The Kaplan-Meier estimator and log-rank test were used to estimate the cumulative probability of the primary and secondary endpoint for patients in the respective ET-1 groups. Since information on mortality following discharge was known in each patient, cases were right-censored at 250 days. Multivariate logistic regression analysis was performed to calculate the odds ratio of the ET-1 high group for predicting the need for ECMO as well as predicting PH. Variables identified as significantly associated with need for ECMO on univariate analysis (defect size, intrathoracic liver position, and PH severity) were included in multivariate logistic regression. Univariate and multivariate analysis with the Cox proportional regression model was used to identify variables independently associated with mortality. Variables that were significantly associated with mortality on univariate analysis (defect size, FETO, left-sided CDH, intrathoracic liver position, severe PH, biventricular cardiac dysfunction, ET-1 high, ventricular disproportion, and ECMO) were included in the multivariate regression. Results are reported as hazard ratio with SE and 95% CI. After the exclusion of colinear variables, only factors that were significantly associated with outcome on univariate analysis were included in multivariate logistic regression. Multicollinearity was tested using the variance inflation factor and Pearson correlation. Goodness of fit was assessed using the Hosmer-Lemeshow test. A p-value of < 0.05 was considered to indicate statistical significance.

## Results

### Patient’s characteristics

Between 2014 and 2022, 221 CDH neonates were treated at our hospital, of which 178 were enrolled in the BIOPTIC study. Of these, 107 CDH newborns were randomly selected for ET-1 analysis based on available blood volume. Twenty patients (18.7%) were born premature (< 37 weeks), and 41 (38.3%) required ECMO. Thirty-seven of these patients received ECMO support, while four had contraindications to ECMO and died within 48 h. The mortality rate in the need for ECMO group was 51.2% (21/41), no device related complications were observed that contributed to mortality. The overall mortality rate was 19.6% (21/107). Therefore, no patient from the non-ECMO group died. Patients’ characteristics in the ECMO and non-ECMO groups are presented in Table 1. The ECMO group had significantly worse CDH characteristics including lower rates of normal cardiac function (*p* < 0.001) and mild/no PH (*p* < 0.001). The median age at first echocardiographic assessment was 3.1 h (IQR 1.8–5.4 h).

**Table 1:** Patients’ characteristics for all patients and within the need for ECMO and no ECMO groups. Data is presented as total number (percentage) or median (IQR). For the comparison of the need for ECMO and no ECMO groups p-values < 0.05 are presented in bold. Not extubated indicates patients who remained intubated at the time of death. CDH, congenital diaphragmatic hernia; ECMO, extracorporeal membrane oxygenation; FETO, fetal endoluminal tracheal occlusion; N/A, not applicable; O/e LHR, observed-to-expected lung-to-head ratio

	Need for ECMO (*n* = 41)	No ECMO (*n* = 66)	*P*-value	All patients (*n* = 107)
**Demographics**				
Gender, male	27 (65.9%)	30 (45.5%)	**0.041**	57 (53.3%)
Gestational age, weeks	38.1 (37.3–38.6)	38.1 (37.4–38.9)	0.175	38.1 (37.4–38.7)
Birth weight, kg	3.2 (2.7–3.4)	3.0 (2.7–3.4)	0.383	3.1 (2.7–3.4)
Inborn	40 (97.6%)	62 (93.9%)	0.39	102 (95.3%)
Prenatally diagnosed CDH	41 (100%)	63 (95.5%)	0.168	104 (97.2%)
Left-sided CDH	32 (78.0%)	62 (93.9%)	**0.015**	94 (88.0%)
o/e LHR, %	40 (33–49)	45 (37–53)	**< 0.001**	40 (33–56)
Intrathoracic liver	36 (87.8%)	23 (34.8%)	**< 0.001**	59 (55.1%)
FETO	10 (24.4%)	5 (7.6%)	**0.015**	15 (14.0%)
**Defect stage**				
A	0	11 (16.7%)	**< 0.001**	11 (10.3%)
B	0	29 (43.9%)	**< 0.001**	29 (27.1%)
C	18 (43.9%)	24 (36.4%)	0.44	42 (39.3%)
D	17 (41.5%)	2 (3%)	**< 0.001**	19 (17.8%)
Not repaired	6 (14.6%)	0	**0.001**	6 (5.6%)
**Outcome**				
Age at ECMO start, h	10.2 (5.9–21.6)	N/A		N/A
ECMO duration, d	7.6 (5.1–18.4)	N/A		N/A
Mechanical ventilation, d	24.4 (14.8–35.8)	7.1 (5.8–9.6)	**< 0.001**	8.9 (6.5–17.1)
Not extubated	16 (39.0%)	0	**< 0.001**	16 (15.0%)
Death	21 (51.2%)	0	**< 0.001**	21 (19.6%)
Time of death, d	35.0 (22.9–62.1)	N/A		N/A
Length of hospital stay, d	35.2 (23.7–64.1)	30.0 (22.1–43.0)	**< 0.001**	35.2 (25.0–65.1)

### Measurements of ET-1 concentration

**Fig. 1:**
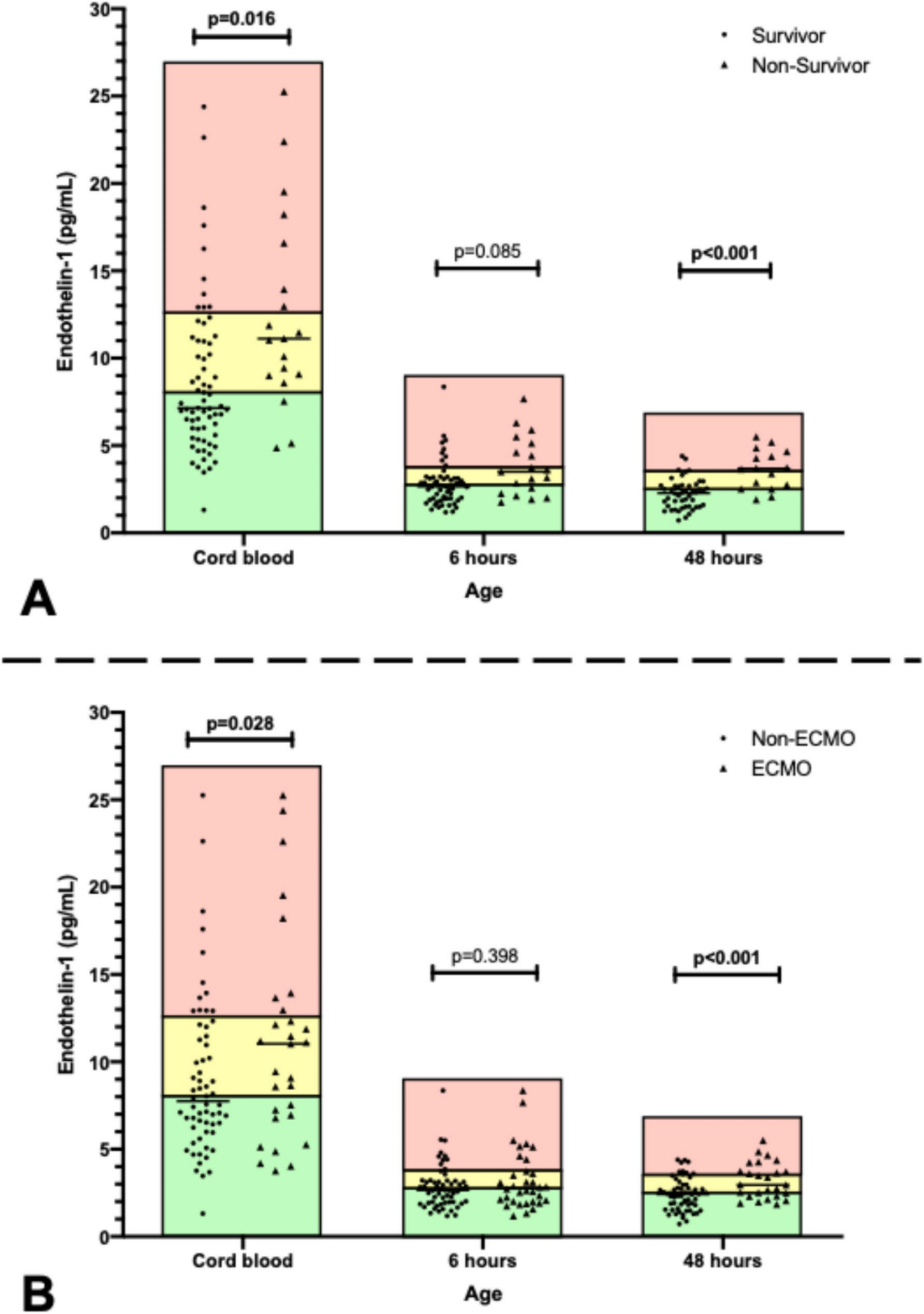
**A**: ET-1 measurements at 0 h, 6 h, 48 h comparing survivors and non-survivors; **B**: ET-1 measurements at 0 h, 6 h, 48 h comparing neonates with and without need for ECMO. The red, yellow, and green boxes represent the high, intermediate, and low ET-1 groups at the respective timepoints

**Fig. 2:**
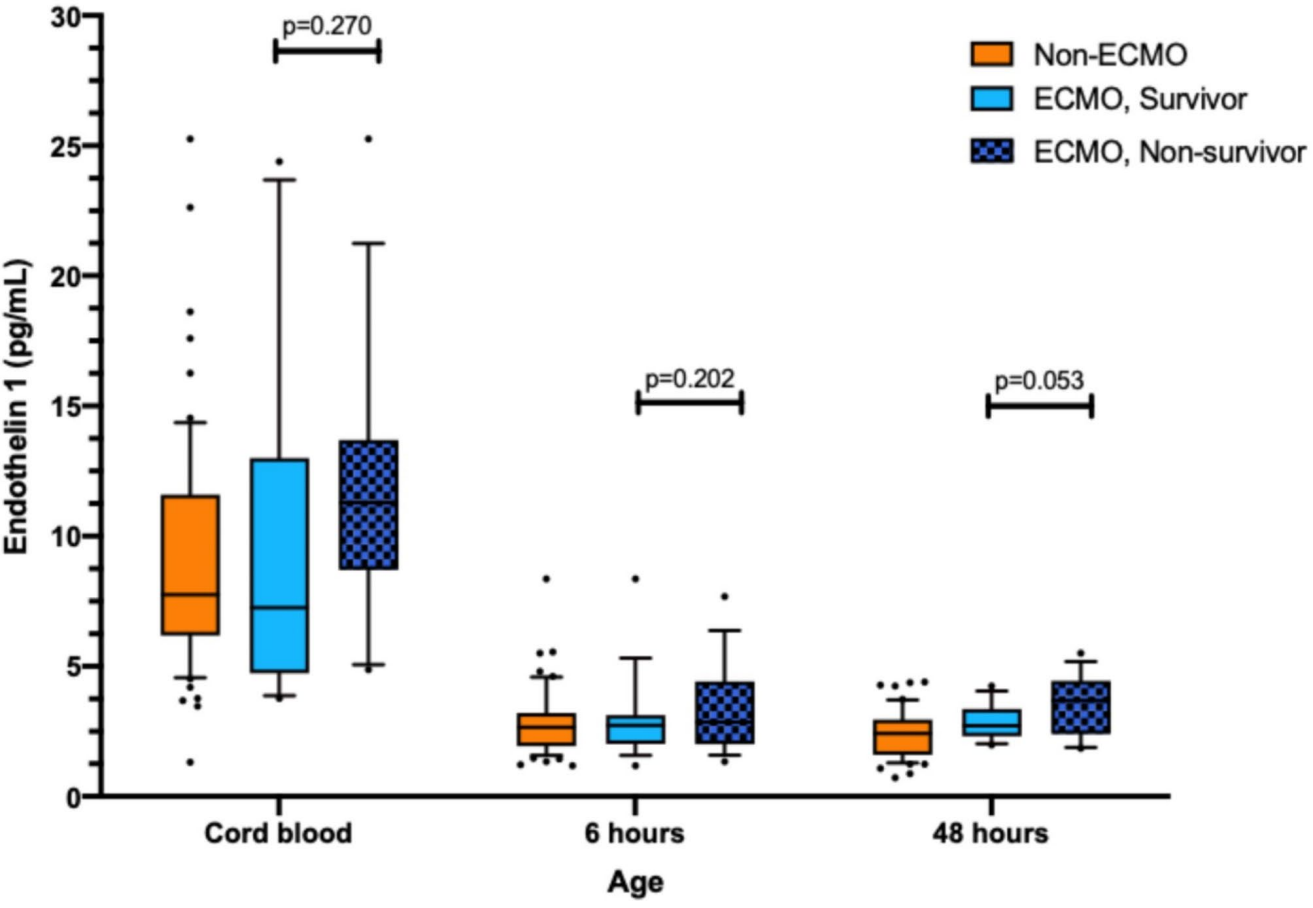
ET-1 measurements at 0 h, 6 h, 48 h comparing neonates without need for ECMO, ECMO survivors, and ECMO non-survivors

ET-1 levels after 0 h, 6 h, and 48 h for the higher cut-off were 12.5 pg/ml, 3.7 pg/ml, and 3.5 pg/ml, respectively. ET-1 levels of 8.0 pg/ml, 2.73 pg/ml, and 2.45 pg/ml, were defined as lower cut-off values, respectively. Median ET-1 values were significantly higher in ECMO versus non-ECMO patients at 0 h (10.24 pg/ml vs. 7.14 pg/ml; *p* = 0.028) and 48 h (2.96 pg/ml vs. 1.90 pg/ml; *p* < 0.001), and comparable at 6 h (2.78 pg/ml vs. 2.69 pg/ml; *p* = 0.398) (Fig. 1A). Similarly, median ET-1 values in non-survivors were significantly higher compared to survivors at 0 h (11.12 pg/ml vs. 7.20 pg/ml; *p* = 0.016) and 48 h (3.71 pg/ml vs. 2.30 pg/ml; *p* < 0.001), and comparable at 6 h (3.18 pg/ml vs. 2.69 pg/ml; *p* = 0.085) (Fig. 1B). Distribution of ET-1 values among non-ECMO patients, ECMO survivors, and ECMO non-survivors are presented in Fig. 2. ET-1 levels in ECMO survivors were not significantly different from ECMO non-survivors at any time point. The distribution of baseline, treatment, and outcome data in the ET-1 group are presented in Table 2. In the ET-1 high group (*n* = 38), 63.2% required ECMO support, and 42.1% died. In the intermediate group (*n* = 44), 27.9% of patients required ECMO and 11.6% died. In the ET-1 low group (*n* = 25), need for ECMO and mortality were 15.4% and 0%, respectively. Data on CDH characteristics, echocardiography, treatment, and outcome correlated with ET-1 group allocation.

**Table 2:** Patients’ characteristics across Endothelin-1 groups. Data is presented as total number (percentage) or median (IQR). P-values < 0.05 are presented in bold. Not extubated indicates patients who remained intubated at the time of death. *Duration of mechanical ventilation and length of hospital stay was only calculated for surviving patients. CDH, congenital diaphragmatic hernia; ECMO, extracorporeal membrane oxygenation; FETO, fetal endoluminal tracheal occlusion; N/A, not applicable; o/e LHR, observed-to-expected lung-to-head ratio

	ET-1 high (*n* = 38)	ET-1 intermediate (*n* = 43)	ET-1 low (*n* = 26)	*P*-value
**Demographics**				
Gender, male	22 (57.9%)	18 (41.9%)	17 (65.4%)	0.131
Gestational age, weeks	37.9 (36.6–39.1)	38.1 (37.6–39.0)	38.3 (37.6–39.1)	0.162
Birth weight, kg	3.0 (2.7–3.4)	3.0 (2.7–3.3)	3.3 (3.0–3.5)	0.11
Left-sided CDH	31 (81.6%)	40 (93%)	23 (88.5%)	0.292
o/e LHR, %	38.0 (30.0–45.0)	40.0 (32.0–49.0)	46.5 (36.5–55.3)	**0.049**
Intrathoracic liver	27 (71.1%)	17 (39.5%)	15 (57.7%)	**0.017**
FETO	8 (21.1%)	6 (14%)	1 (3.8%)	0.153
Bosentan	15 (39.5%)	8 (18.6%)	1 (3.9%)	**0.003**
**Defect stage**				
A	1 (2.6%)	6 (14%)	4 (15.4%)	0.154
B	5 (13.2%)	15 (34.9%)	9 (34.6%)	0.057
C	19 (50%)	12 (27.9%)	11 (42.3%)	0.121
D	9 (23.7%)	8 (18.6%)	2 (7.7%)	0.258
Not repaired	4 (10.5%)	2 (4.7%)	0	0.19
**Outcome**				
ECMO	24 (63.2%)	13 (30.2%)	4 (15.4%)	**< 0.001**
Age at ECMO start, h	8.8 (6.0–9.7)	13.9 (5.3–31.6)	30.0 (25.9–31.6)	0.094
ECMO duration, d	13.1 (5.8–22.1)	6.3 (4.9–9.5)	4.4 (3.4–5.4)	0.055
Mechanical ventilation, d*	10.9 (6.8–22.2)	7.3 (5.5–11.3)	9.1 (6.8–11.0)	0.078
Not extubated	12 (31.6%)	4 (9.3%)	0	**0.001**
Death	16 (42.1%)	5 (11.6%)	0	**< 0.001**
Time of death, d	34.2 (18.5–55.2)	35.2 (14.3–47.6)	N/A	0.53
Length of stay, d*	43.0 (34.1–80.0)	28.3 (21.5–58.0)	41.6 (23.6–50.2)	0.061
**ET-1**				
0 h	12.9 (7.9–17.3)	8.5 (7.1–10.1)	5.5 (4.7–6.6)	**< 0.001**
6 h	3.4 (2.8–3.9)	2.5 (1.9–3.0)	2.0 (1.6–2.0)	**< 0.001**
48 h	3.0 (2.4–3.2)	2.44, (1.89, 2.69)	1.33, (1.25, 1.57)	**< 0.001**

### Outcome prediction in CDH neonates

**Fig. 3:**
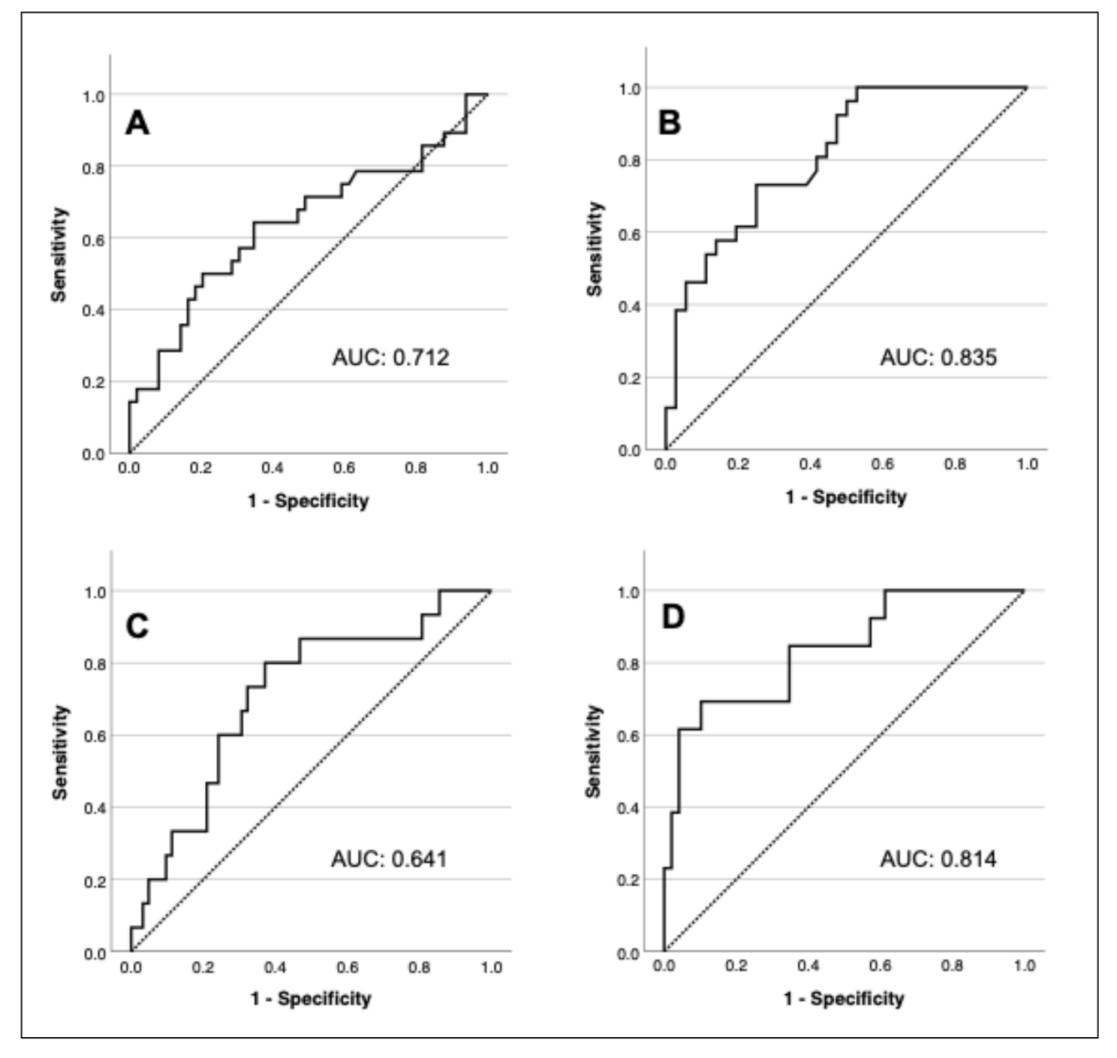
Receiver operating characteristics curves predicting need for ECMO and mortality from cord blood **(A+C)** and at 48 h **(B+D)**. AUC, area under the curve

Using ROC analysis, the AUC for predicting need for ECMO at 0 h, 6 h, and 48 h was 0.641 (95% CI 0.505–0.777, *p* = 0.043), 0.557 (95% CI 0.424–0.691, *p* = 0.399), and 0.814 (CI 0.710–0.917), *p* < 0.001), respectively (Fig. 3A-B). AUC for survival at those time points was 0.712 (95% CI 0.568–0.855, *p* = 0.004), 0.645 (95% CI 0.483–0.807, *p* = 0.079), and 0.835 (95% CI 0.714–0.955, *p* < 0.001) (Fig. 3C-D). At 48 h, 34 patients (31.8%) required ECMO, and 3 (2.8%) had died.

**Fig. 4:**
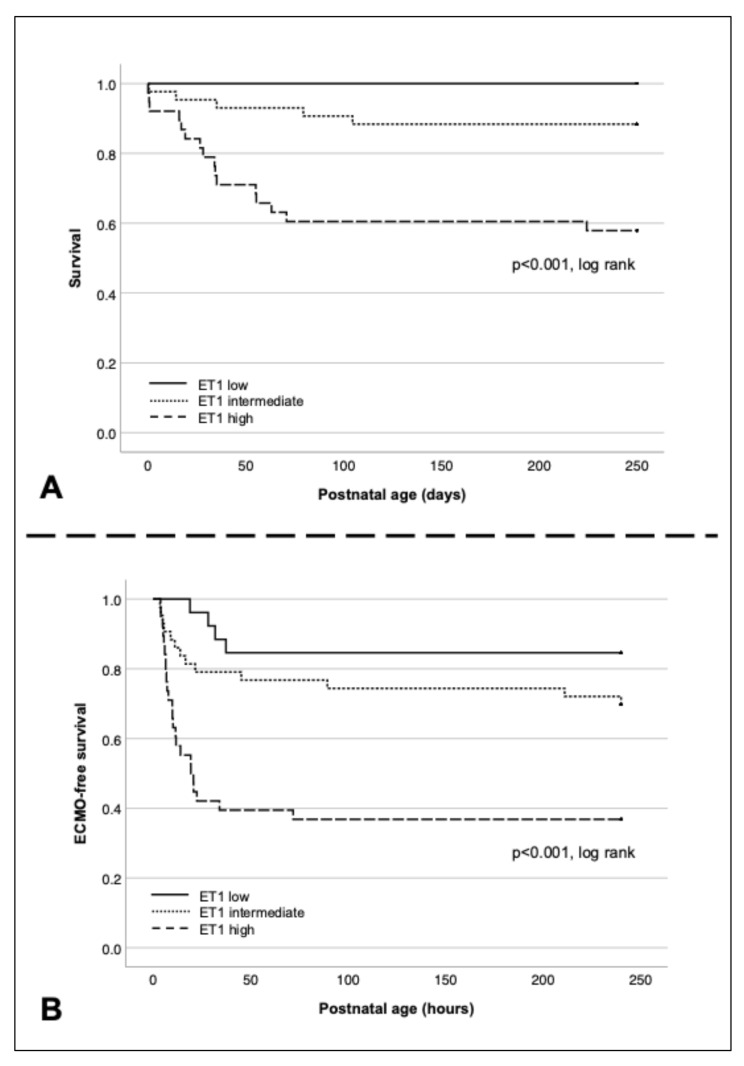
**A**: Kaplan-Meier curves for survival comparing high, intermediate, and low ET-1 groups; **B**: Kaplan-Meier curves for ECMO-free survival comparing high, intermediate, and low ET-1 groups

Kaplan-Meier survival curves and ECMO-free survival curves for the ET-1 high, intermediate, and low groups are displayed in Fig. 4. Cumulative survival through day 250 was highest in the ET-1 low group and lowest in the ET-1 high group (log-rank, *p* < 0.001). Similarly, ECMO-free survival was highest in the ET-1 low group and lowest in the ET-1 high group (log-rank, *p* < 0.001).

### Uni- and multivariate regression analysis

On multivariate analysis, ECMO use was significantly associated with defect size (OR: 6.29; 95% CI: 1.29–30.74; *p* = 0.023), intrathoracic liver position (OR: 16.3; 95% CI: 1.71–155.15; *p* = 0.015), and PH severity (OR: 8.93; 95% CI: 1.57–45.0; *p* = 0.013). ET-1 high was not independently associated with need for ECMO (OR: 1.99; 95% CI: 0.32–12.61; *p* = 0.461).

Cox regression identified defect size, FETO, left-sided CDH, intrathoracic liver position, severe PH, biventricular cardiac dysfunction, ET-1 high, ventricular disproportion, and ECMO as significantly associated with mortality in the univariate analysis. Sex, gestational age, non-isolated CDH, birth weight, patch repair, o/e LHR, and cesarean section were not significantly associated and were therefore not included in the multivariate analysis. Defect size, biventricular cardiac dysfunction, and ECMO remained independently associated with mortality on multivariate regression (Table 3). Univariate logistic regression showed significant associations between severe PH and o/e LHR (OR: 0.96; 95% CI: 0.92–0.99; *p* = 0.038), intrathoracic liver position (OR: 2.9; 95% CI: 1.2–7.3; *p* = 0.024), biventricular dysfunction (OR: 9.1; 95% CI: 3.5–23.7; *p* < 0.001), and ET-1 high (OR: 5.9; 95% CI: 2.3–14.9; *p* < 0.001). Multivariate analysis confirmed biventricular dysfunction (OR: 6.2; 95% CI: 2.1–18.2; *p* = 0.001), and ET-1 high (OR: 4.8; 95% CI: 1.6–13.8; *p* = 0.004) as independently associated with severe PH.

**Table 3:** Cox regression survival analysis. Variables significantly associated with survival on univariate analysis were further analyzed in multivariate analysis. P-values < 0.05 are presented in bold. BV, biventricular; CDH, congenital diaphragmatic hernia; CI, confidence interval; ECMO, extracorporeal membrane oxygenation; ET-1, endothelin-1; FETO, fetal endoluminal tracheal occlusion; HR, hazard ratio; O/e LHR, observed-to-expected lung-to-head ratio; PH, pulmonary hypertension; SE, standard error

Variable	Univariate Analysis				Multivariate Analysis			
	HR	95% CI	SE	*P*-value	HR	95% CI	SE	*P*-value
Sex	1.12	0.48–2.65	0.44	0.79				
Gestational age, weeks	0.89	0.69–1.14	0.13	0.34				
Non-isolated CDH	1.01	0.35–2.93	0.54	0.99				
Birth weight, kg	0.67	0.27–1.67	0.47	0.39				
**Defect size**	**5.92**	**3.00–11.65**	**0.35**	**< 0.001**	**2.68**	**1.18–6.08**	**0.42**	**0.02**
Patch repair	3.05	0.39–23.79	1.05	0.29				
FETO	**2.53**	**1.01–6.35**	**0.47**	**0.05**	2.11	0.55–8.15	0.69	0.28
Left-sided CDH	**3.23**	**1.29–8.08**	**0.47**	**0.01**	2.46	0.71–8.54	0.63	0.16
Liver-up	**43.60**	**1.11–177.71**	**1.87**	**0.04**	105.77	0.00–421.00	112.70	0.92
o/e LHR	1.00	0.96–1.04	0.02	0.91				
Severe PH	**6.39**	**2.32–17.60**	**0.52**	**< 0.001**	1.71	0.43–6.83	0.71	0.45
**BV cardiac dysfunction**	**7.67**	**2.58–22.85**	**0.56**	**< 0.001**	**11.43**	**1.49–87.90**	**1.04**	**0.02**
ET-1 high	**4.43**	**1.61–12.19**	**0.52**	**0.00**	2.59	0.64–10.51	0.72	0.18
C-section	1.43	0.56–3.68	0.48	0.46				
Ventricular disproportion	**3.58**	**1.30–9.89**	**0.52**	**0.01**	2.87	0.78–10.63	0.67	0.11
**ECMO**	**3.77**	**1.21–11.72**	**0.58**	**0.02**	**9.86**	**1.34–72.42**	**1.02**	**0.02**

### PH on follow-up echocardiogram and ET-1 antagonist treatment

Follow-up echocardiogram data on PH severity were available for 104 patients (97.2%) at a median of 12.6 days (IQR: 11.1–14.7 days). Mild/no PH, moderate PH, and severe PH were present in 62.0%, 21.3%, and 13.0% of patients, respectively. PH severity at follow-up was significantly higher in the ET-1 intermediate and high group (Table 4). Bosentan treatment was administered to 3.8%, 18.6%, and 39.5% of patients in the ET-1 low, intermediate, and high groups (*p* = 0.003), respectively. Bosentan treatment was started at a significantly younger age in the ET-1 high group compared to the low and intermediate groups (median 9.2 vs. 24.0 days, *p* = 0.017). Among patients with moderate or severe PH at follow-up, 81.1% of those receiving bosentan improved by at least one PH severity grade compared to 26.7% without bosentan (*p* = 0.004).

**Table 4:** Initial PH, initial cardiac dysfunction, and follow-up PH assessed using echocardiography; Bosentan treatment; group comparison across ET-1 groups using Chi^2^. P-values < 0.05 are presented in bold. BV, biventricular; ET-1, Endothelin-1; LV, left ventricular; PH, pulmonary hypertension; RV, right ventricular

	ET-1 high (*n* = 38)	ET-1 intermediate (*n* = 43)	ET-1 low (*n* = 26)	*P*-value
**Initial PH**				
Mild/ no PH	13.2% (*n* = 5)	37.2% (*n* = 16)	50.0% (*n* = 13)	**< 0.001**
Moderate PH	36.8% (*n* = 14)	41.9% (*n* = 18)	46.2% (*n* = 12)	
Severe PH	50.0% (*n* = 19)	20.9% (*n* = 9)	3.4% (*n* = 1)	
**Initial Cardiac function**				
Normal	26.3% (*n* = 10)	41.9% (*n* = 18)	53.8% (*n* = 14)	0.127
RV dysfunction	28.9% (*n* = 11)	23.3% (*n* = 10)	34.6% (*n* = 9)	
LV dysfunction	2.6% (*n* = 1)	4.7% (*n* = 2)	0.0% (*n* = 0)	
BV dysfunction	42.1% (*n* = 16)	30.2% (*n* = 13)	11.5% (*n* = 3)	
RV/LV ratio > 1.1	57.9% (*n* = 22)	39.5% (*n* = 17)	23.1% (*n* = 6)	**0.02**
**Bosentan treatment**	39.5% (*n* = 15)	18.6% (*n* = 8)	3.8% (*n* = 1)	**0.003**
**Follow-up PH**				
Mild/ no PH	30.6% (*n* = 11)	73.8% (*n* = 31)	96.2% (*n* = 25)	**< 0.001**
Moderate PH	41.7% (*n* = 15)	16.7% (*n* = 7)	3.8% (*n* = 1)	
Severe PH	27.8% (*n* = 10)	9.5% (*n* = 4)	0.0% (*n* = 0)	

## Discussion

This is the first investigation of serial ET-1 levels in the first 48 h of life in CDH in relation to disease severity and outcomes. We observed an association between high ET-1 levels with significantly higher rates of need for ECMO and mortality in this population. Elevated ET-1 levels were additionally associated with more severe disease characteristics, including a higher prevalence of severe PH and biventricular dysfunction. ET-1 levels remained significantly associated with severe PH in multivariate analysis. Notably, ROC analysis demonstrated that ET-1 levels at 48 h had the highest predictive value of the three timepoints for both need for ECMO and mortality. Furthermore, follow-up echocardiogram data revealed that PH severity was significantly higher in patients with high ET-1 levels. Although physicians were unaware of individual ET-1 levels at the time of echocardiography assessment, bosentan treatment was more commonly used in the ET-1 high group and associated with significant PH improvement.

ET-1, the predominant isoform of the peptide Endothelin [15], binds to ET_A_ and ET_B_ receptors [16], playing a crucial role in the vasoactive pathway of the pulmonary vascular system, among other areas of the body [17]. ET_A_ receptors are found predominantly on vascular smooth muscle cells (SMCs) and ET_B_ receptors are present on both endothelial cells and vascular SMCs [15]. Activation of ET_A_ on SMCs causes vasoconstriction, while activation of ET_B_ on endothelial cells promotes vasodilation [18]. In fetal life, ET-1 is regulated primarily by the hypoxic conditions of the intrauterine environment and supports the maintenance of a high pulmonary vascular resistance necessary for fetal circulation. As the fetus approaches term, ET-1 levels and receptor activity begin to adapt in preparation for the transition to postnatal life. The placenta also significantly contributes to ET-1 levels in the fetal circulation, regulating systemic vascular resistance and ensuring normal uteroplacental blood flow. Placental dysfunction, as seen in preeclampsia, disrupts this regulation, leading to higher ET-1 levels [19, 20] and increased vascular resistance in both the fetus and mother. However, placental dysfunction was not observed in our cohort. At delivery, the transition from the intrauterine to extrauterine environment leads to rapid changes in ET-1 regulation [21, 22]. With the onset of breathing and aeration of the lungs, pulmonary vascular resistance drops while oxygenation increases, leading to downregulation of ET-1 levels in the pulmonary circulation. This decrease is paramount for the vasodilation needed to establish normal pulmonary blood flow. ET-1 is also involved in modulating systemic vascular resistance after the neonate is liberated from placental blood flow. This phenomenon explains the notable decrease in median ET-1 levels across all patients from 0 to 6 h, with ET-1 levels remaining stable at 48 h, which reflects the physiological changes following cord clamping [23].

In a rat model, the pulmonary vasoconstrictive response to ET-1 was greater in nitrofen-induced CDH rats compared to controls [24]. Another study reported elevated ET-1 levels in newborns with PH, with a shift in receptor expression favoring ET_A_, further contributing to pulmonary vasoconstriction [16, 25]. These findings suggest that pathological alterations in the endothelin system in CDH may include increased binding of ET-1 to a higher number of ET_A_ receptors, combined with fewer ET_B_ receptors available. Our study supports these findings demonstrating elevated ET-1 levels in newborns with adverse outcomes, although we were not able to directly assess potential imbalances between ET_A_ and ET_B_ receptors.

In a landmark study by Keller et al. elevated ET-1 levels during the first two weeks of life were associated with poor outcome in CDH neonates, defined as death or discharged on oxygen [9]. That study additionally demonstrated a strong correlation between PH severity on echocardiogram and ET-1 levels [9]. Severity of PH is one of the main pathophysiological determinants of adverse outcome in CDH neonates [7]. The previously described association of dysregulated ET-1 and PH severity in CDH neonates was also observed in our study and may be a contributing mechanism to the relationship with increased mortality and need for ECMO.

Although ET-1 levels were significantly different between groups at 0 and 48 h, this was not observed at 6 h. This may be explained by the physiological changes after birth [23], which could affect ET-1 levels more acutely at 6 h compared to 48 h. The heterogeneity of the clinical course and the severity of cardiorespiratory failure varies in CDH neonates, especially in the first 12 h of life and even between patients with comparable CDH severity. Also, a certain honeymoon period is frequently observed with an initially stable phase leading to subsequent deterioration with the need for ECMO support. Leyens et al. demonstrated that PH severity in CDH newborns is dynamic during the first 48 h of life, further supporting the hypothesis that early physiological changes influence biomarker levels [26]. It is also notable that 24.4% of patients requiring ECMO during the study period were receiving it by 6 h, which may have affected ET-1 plasma concentration due to dilution within the ECMO circuit. However, a thorough analysis did not reveal any correlation with the timing of ECMO support and ET-1 levels. The wide dispersion of ET-1 levels observed in umbilical cord blood, as shown in Figs. 1 and 2, might also reflect transitional physiological changes occurring immediately after birth. This variability may explain the lower predictive capacity of ET-1 levels in cord blood compared to measurements at later time points.

Bosentan, an ET-1 receptor antagonist, is an available adjunctive therapy in neonates with CDH. By blocking ET-1 receptors, bosentan reduces vasoconstriction [8, 27]. In our hospital, bosentan is administered, when PH exceeds 2/3 of systemic pressure, despite the use of iNO or sildenafil, in the presence of a persistently high oxygen demand or an oxygenation index (OI) greater than 15. In this study the increasing rate of bosentan use from the ET-1 low to intermediate and high groups again supports a correlation between ET-1 levels and PH severity and support the promising role of the ET-1 pathway as a therapeutic target in CDH. However, recent studies suggest that CDH phenotypes vary, requiring tailored therapies [6, 28]. For example, patients with significant post-capillary PH may benefit less from pulmonary vasodilators and may require inotropes to improve left ventricular function [28]. Future research is required to investigate the differences in ET-1 levels across CDH to identify those who would benefit most from targeting the ET-1 pathway.

This study has some limitations. First, blood was drawn from the umbilical vein, followed by sampling from an arterial line, due to practical clinical reasons. Second, clinical management may have evolved over the recruitment period, although the use of pulmonary vasodilators in the first 48 h remained similar over this time. Third, the impact of other components of CDH disease pathophysiology on ET-1 expressions, over and above other neonatal PPHN states on ET-1 expression, is incompletely understood. However, given the potential available treatment options of the ET-1 pathway, these findings deserve further investigation. Forth, the time between the initial echocardiographic assessment within the first six hours of life and ET-1 measurements at 48 h might limit accurate conclusions about the association with PH severity and ET-1 at that later time point, but we did not have routinely performed echocardiography studies available in all patients at 48 h. Finally, although the fact that patients were included in this study based on the availability of a sufficient amount of plasma for ET-1 measurements might be associated with a certain selection bias, the distribution of baseline characteristics in this cohort represent the heterogeneity of CDH presentation after birth, making this cohort comparable to other previously published studies.

In conclusion, elevated ET-1 levels in CDH newborns are associated with worse clinical outcomes, with a particularly strong association with PH, exceeding its predictive value for mortality and need for ECMO. These results underscore ET-1’s potential as a prognostic biomarker and therapeutic target in this population, with potential implications when measured within the first 48 h of life. The identification of ET-1 as a biomarker in neonates with CDH is particularly important given the limited availability of well-established prognostic markers in this population. By tailoring the use of pulmonary vasodilators based on ET-1 levels, clinicians could potentially optimize treatment strategies.

## Data Availability

The datasets used and/or analysed during the current study are available from the corresponding author on reasonable request.

## Abbreviations

AUC: Area Under the Curve
ASE: American Society of Echocardiography
BV: Biventricular
CI: Confidence Interval
CDH: Congenital Diaphragmatic Hernia
ET-1: Endothelin-1
ETA: Endothelin receptor type A
ETB: Endothelin receptor type B
ECMO: Extracorporeal Membrane Oxygenation
FETO: Fetal Endoscopic Tracheal Occlusion
iNO: Inhaled Nitric Oxide
IQR: Interquartile Range
LV: Left ventricular
LVD: Left Ventricular Diameter
NICU: Neonatal Intensive Care Unit
o/e LHR: Observed/Expected Lung-to-Head Ratio
OI: Oxygenation index
OR: Odds Ratio
PAP: Pulmonary Artery Pressure
PH: Pulmonary Hypertension
RV: Right ventricular
RVD: Right Ventricular Diameter
SE: Standard Error
